# Dead Pericarps of Dry Fruits Function as Long-Term Storage for Active Hydrolytic Enzymes and Other Substances That Affect Germination and Microbial Growth

**DOI:** 10.3390/plants6040064

**Published:** 2017-12-19

**Authors:** James Godwin, Buzi Raviv, Gideon Grafi

**Affiliations:** French Associates Institute for Agriculture and Biotechnology of Drylands, Blaustein Institutes for Desert Research, Ben-Gurion University of the Negev, Midreshet Ben Gurion 84990, Israel; godwin.j93@gmail.com (G.J.); buzi@post.bgu.ac.il (B.R.)

**Keywords:** dead organs, dry fruits, pericarps, hydrolytic enzymes, nucleases, chitinases, proteases, germination, microbial growth, *Sinapis alba*

## Abstract

It is commonly assumed that dead pericarps of dry indehiscent fruits have evolved to provide an additional physical layer for embryo protection and as a means for long distance dispersal. The pericarps of dry fruits undergo programmed cell death (PCD) during maturation whereby most macromolecules such DNA, RNA, and proteins are thought to be degraded and their constituents remobilized to filial tissues such as embryo and endosperm. We wanted to test the hypothesis that the dead pericarp represents an elaborated layer that is capable of storing active proteins and other substances for increasing survival rate of germinating seeds. Using in gel assays we found that dead pericarps of both dehiscent and indehiscent dry fruits of various plant species including *Arabidopsis thaliana* and *Sinapis alba* release upon hydration multiple active hydrolytic enzymes that can persist in an active form for decades, including nucleases, proteases, and chitinases. Proteomic analysis of indehiscent pericarp of *S. alba* revealed multiple proteins released upon hydration, among them proteases and chitinases, as well as proteins involved in reactive oxygen species (ROS) detoxification and cell wall modification. Pericarps appear to function also as a nutritional element-rich storage for nitrate, potassium, phosphorus, sulfur, and others. *Sinapis alba* dehiscent and indehiscent pericarps possess germination inhibitory substances as well as substances that promote microbial growth. Collectively, our study explored previously unknown features of the dead pericarp acting also as a reservoir of biological active proteins, and other substances capable of “engineering” the microenvironment for the benefit of the embryo.

## 1. Introduction

Angiosperm evolution has led to the development of diverse fruit morphologies to assist seed dispersal [1]. While the seed is the fundamental unit of dispersal in higher plants, plants have evolved a variety of dispersal units commonly thought to provide embryos with a physical protective layer(s) and means for dispersal in the habitat [1,2]. Dry fruits consist of two major groups, dehiscent in which the fruit splits open at maturity to allow for seed dispersal, and indehiscent whereby the fruit is not opened at maturity and constitutes the dispersal unit. The pericarp is developed from the ovary wall or ovary wall, plus some accessory parts and this region comprises of an outer layer, the exocarp, the middle layer, the mesocarp, and the inner layer, the endocarp, which is distinct in fleshy fruits; in dry fruits, the subdivisions of the pericarp are not clear [3].

During seed development, maternal tissues enclosing the embryo such as the nucellus, the seed coat, the pericarp, and the nucellar projections undergo a progressive degeneration by programmed cell death (PCD). As a result macromolecules such as DNA, RNA and proteins are degraded and their constituents remobilized to filial tissues like endosperm and the embryo [4].

Molecular and biochemical studies on the role played by the dead pericarp of dry indehiscent fruits are limited. However, multiple studies on the developmental and physiological function of the pericarp suggested involvement in seed dormancy, germination and seedling establishment [5,6,7,8,9]. In general, pericarps inhibit seed germination by reducing water uptake by seeds, limiting gaseous exchange, releasing germination inhibitors, or mechanically by restricting embryo growth and preventing radicle protrusion [10,11,12,13]. In dry years, the presence of the pericarp might provide a protective layer that reduces desiccation rates of seeds [14,15]. The study of *Cryptocarya alba* pericarp on seedling recruitment and biomass showed that the pericarp showed an effect on the number of seeds that are recruited, but not on the biomass that seedlings attain [14]. The pericarp may also provide the means (e.g., spines, hooks, barbs) for long distance dispersal [16].

A recent study demonstrated that dead organs enclosing the embryo both in dicots and monocots (e.g., seed coats, glumes in grasses) store and release upon hydration hydrolytic enzymes and other substances that might affect seed biology [17,18]. Based on these findings, we sought to investigate the capacity of dead pericarps of dehiscent and indehiscent fruits in storing and releasing hydrolytic enzymes and other substances following hydration and their role in seed germination and microbial growth. To this end, we selected various plant species (e.g., Brassicaceae and Fabaceae species) including the model plant *Arabidopsis thaliana*, *Spartium junceum* (both possess dehiscent fruits) as well as *Sinapis alba* whose fruit has two distinguishable parts, the lower part, proximal to the pedicel containing 2–4 seeds, which is provisionally dehiscent and can be easily opened by modest pressure (will be referred to as dehiscent part) and the distal indehiscent beak that contains a single seed. Our data showed that dead dehiscent and indehiscent pericarps store and release upon hydration active hydrolases including nucleases, proteases and chitinases that can persist in active form for decades. Furthermore, pericarps were found to store high levels of nutrients including nitrate (NO_3_), potassium (K) and sulfur (S). Pericarps also contain germination inhibitors and bacterial growth promoting substances that could have an effect on seed persistence in the soil, germination and seedling establishment.

## 2. Methodology

### 2.1. Plant Material and Growth Conditions

Seeds and fruits of various wild, cultivated and ornamental plant species were collected in the field in Israel or purchased from the local market (Supplemental Table S1). Seeds of *Brassica rapa* were purchased from Wisconsin FASTPLANTS (https://fastplants.org/). This is a variety developed through a long-term artificial selection plant-breeding program at the University of Wisconsin-Madison. Pericarps of *Arabidopsis thaliana* (Col) were collected from plants grown in growth room at 22 °C ± 2 °C under long day photoperiod. Dry, dead pericarps were ground and stored at −20 °C until used.

### 2.2. In-Gel Nuclease Assay

Nuclease assay was performed essentially as described [19] in polyacrylamide gel containing 300 μg/mL denatured salmon sperm DNA or ribonucleic acid from Torula yeast (Sigma, St. Louis, MO, USA) for RNases activity. Briefy, 10 mg ground pericarps were incubated in 100 μL of PBS at 4 °C for 16 h, after which the supernatant was collected and proteins (equivalent to 2.5 mg pericarps) were incubated with Sample Buffer for 1 h at 37 °C followed by separation on 12% sodium dodecyl sulfate polyacrylamide gel electrophoresis (SDS-PAGE). The gel was washed twice, at 30 min at room temperature in buffer containing 10 mM Tris-HCl pH 7.5 and 25% isopropanol, followed by washing twice 15 min each with 10 mM Tris-HCl pH 7.5. Nuclease activity was performed by incubating the gel with 10 mM Tris-HCl pH 7.5 containing divalent cations (10 mM MgSO_4_, 10 mM CaCl_2_) for 75 min at 37 °C. The gel was stained for 60–80 min with 10 mM Tris HCl pH 7.5 containing 2 μg/mL ethidium bromide and inspected under ultra-violet (UV) light.

### 2.3. In-Gel Chitinase Assay

In gel chitinase assay was performed essentially as described [20]. Briefly, 10 mg ground pericarps were incubated in 100 μL 0.1 M NaHPO_4_ (pH 6) at 4 °C for 16 h, after which the supernatant was collected and released proteins (equivalent to 2.5 mg pericarps) were separated on 12% SDS-PAGE. Samples (in some experiments samples were concentrated by lyophilization) were first incubated in chitin sample buffer (15% sucrose, 2.5% SDS, 12.5 mM Tris-HCl pH 6.7, 0.01% Bromophenol Blue) for 1 h at 37 °C and samples were run on 12% SDS-PAGE containing 0.01% glycol chitin. The gel was incubated in buffer containing 100 mM sodium acetate (pH = 5.2) and 1% triton X-100 for 2 h at 37 °C followed by staining for 5 min with 0.01% calcofluor white in 500 mM Tris-HCl (pH = 8.9). The gel was washed with distilled water for 1 h and visualized by UV transillumination.

### 2.4. In-Gel Protease Assay 

In-gel protease assay was performed essentially as described [21]. Briefly, 10 mg of ground pericarps were incubated in 100 μL in PBS at 4 °C for 16 h, after which the supernatant was collected and proteins (equivalent to 2.5 mg pericarps) were separated on SDS/PAGE. Samples were first incubated in loading gel sample buffer for 1 hr at 37 °C, samples were loaded and run in 12% SDS-PAGE containing 0.12% gelatin. After running was completed, the gel was washed twice each 45 min in buffer containing 10 mM Tris-HCl (pH = 7.5) and 0.25% Triton x-100 followed by overnight incubation in 10 mM Tris-HCl (pH = 7.5). The gel was then incubated in 10 mM Tris-HCl (pH = 7.5) containing 10 mM CaCl_2_ and 10 mM MgCl_2_ for 30 min at 30 °C and stained by Coomassie blue for 1 h at room temperature.

### 2.5. Proteome Analysis

Proteome analysis of *S. alba* indehiscent pericarps was performed by the proteomic services of The Smoler Protein Research Center at the Technion, Israel. Proteins released from 10 mg of *S. alba* ground pericarps (4 °C, 12 h in phosphate-buffered saline (PBS)) were digested with trypsin followed by separation and mass measurement on LC-MS/MS on LTQ-Orbitrap. Mass spectrometry proteomics profiling and initial processing of the results with Discoverer 1.4 against Uniprot *Brassiceceae* proteins were carried out at the Smoler Proteomics Center of the Technion. All the identified peptides were filtered with high confidence, top rank, mass accuracy, and a minimum of two peptides. High confidence peptides were passed the 1% FDR threshold (FDR = false discovery rate, is the estimated fraction of false positives in a list of peptides). Two replicates were performed and semi-quantitation was done by calculating the peak area of each peptide. The area of the protein was calculated from the average of the two to three most intense peptides from each protein.

Further data analysis was carried out by Vered Chalifa-Caspi, Bioinformatics Core Facility, NIBN, BGU. It is a common practice to require a minimum of two peptides per protein per sample in order to consider the protein as present in that sample. However, we wanted to set a more stringent cutoff for large proteins. Therefore, considering two peptides as the minimum requirement for proteins of length 400 amino acids, we extrapolated to cutoff such that the minimum number of peptides per protein is the protein length divided by 200 (i.e., 400/2), but not less than two peptides. In addition, we requested that the protein coverage by peptides from all samples will be higher than 10% or, in a separate analysis, more than 20%. A protein was considered present if it was present in at least two replicate samples. Proteome parameter definition are given in supplementary materials.

Protein names, their InterPro domains and gene ontology (GO) annotations were retrieved from UniProt. GO ontology files (in .obo format) were downloaded from the Gene Ontology web site (http://geneontology.org/page/download-ontology). GO categorization was carried out using BiNGO (v3.0.3) in Cytoscape (v3.4.0).

### 2.6. Nutrient Analysis

For nutrient analysis, 10 mg of ground *S. alba* and *A. thaliana* pericarps and 10 mg of *S. alba* seed coats were incubated in 300 µL ultrapure Milli-Q water at cooled orbital shaker (4 °C). Analysis of nutrients released from seeds was performed as follows: 20 seeds of *S. alba* and 10 mg of *A. thaliana* seeds were incubated in 600 and 300 µL, respectively, of ultrapure milli-Q water. All samples were incubated for 12 h at 4 °C followed by centrifugation (14,000 rpm, 4 °C), the supernatant was collected and filtered using 0.22 μm spin filter. 200 μL of the filtered supernatant were diluted with 4.8 mL of ultrapure Milli-Q water and subjected to nutrient analysis. Three measurements were performed for each chemical element. The content of macro- and microelements released upon hydration was determined by inductively coupled plasmaoptical emission spectroscopy (ICP-OES) using ICP-720-ES (Varian Inc., Palo Alto, CA, USA). Soluble anions were detected and identified by Ion Chromatography (IC). IC was performed by an ICS-5000 instrument (Dionex, Thermo Fisher Scientific, Waltham, MA, USA). Obtained data were analyzed by Chromeleon 6.8 chromatography data system (Dionex, Thermo Fisher Scientific).

### 2.7. Antibacterial Assay

The assay was performed essentially as described [22]. Briefly, *Escherichia coli* (ATCC 10978) were grown overnight on Lysogeny broth (LB) nutrient broth at 37 °C, the culture was diluted, transferred to 25% LB broth and grown at 37 °C to 0.03–0.05 optical density (OD_595_; Epoch, Biotek, Winooski, VT, USA). A 150 μL aliquot of the culture was incubated with 50 μL of PBS, 50 μL of PBS + 25% Hoagland solution, kanamycin (final concentration 50 μg/mL) or with 50 μL filtered (through 0.2 μm) pericarp extract (three replicates per treatment) in a flat-bottom 96-well microtiter plate. Plates were incubated in the dark using a spectrophotometer (Synergy 4, Biotek, USA) and reads (OD_595_) were taken in intervals of 30 min in a course of 24 h. The average OD for each blank replicate at a given time point was subtracted from the OD of each replicate treatment at the corresponding time point and standard errors were calculated for each treatment at every time point.

### 2.8. Seed Germination Assays

Field experiments were performed on loessial soil in the northern Negev (31°26′57″ N/34°39′31″ E, elevation 176 m) using a 36 square grid (4 × 9), each square is 3 × 3 cm. 18 seeds or seeds with pericarps or 18 beaks containing seeds of *S. alba* were placed in each grid and covered with 1 cm of loessial soil on 9 September 2016. Germination was inspected and scored on 16 December 2016. During this period of time 52 mm precipitation were measured in the abovementioned area (Ruhama meteorological station, https://ims.data.gov.il/he/ims-results).

Lab germination experiments were performed as follows: The germination assay was based on the “cigar roll” method [23], 18 uniform-sized seeds derived from the *S. alba* indehiscent part of the fruit (beak) or 12 dispersal units (indehiscent beak) were placed on moist germination paper (25 cm × 38 cm; Anchor Paper Co., St. Paul, MN, USA), about 4 cm apart, 4 cm below the edge of the paper. The paper was covered with another sheet of moist germination paper and the sandwich was rolled to a final diameter of 2 cm. The base of each roll was placed in a glass beaker containing distilled water for three weeks.

For germination inhibitors test, experiments were carried out in petri plates embedded with Whatman filter paper Grade 1 and irrigated with distilled water or indehiscent pericarp extracts at various concentrations (10, 20, 40, 80, 100 mg/mL). The indehiscent pericarp extract was prepared by incubation of ground pericarp in distilled water for 4 h at 4 °C followed by centrifugation at high speed (10 min, 4 °C, 12,000 rpm), the supernatants were collected and used for the above mentioned experiments (three replicates). The plates were maintained at growth room conditions (22 °C ± 2 °C under long day photoperiod). Final germination percentages were calculated as the mean of three replicates (±standard deviation, SD), while recovery germination percentages according to the following equation [24]:

Recovery germination percentage = {[(a − b)/(c − b)] × 100}(1)

where “a” is the total number of seeds germinated in the pericarp extract solutions plus those that recovered germination in the distilled water, “b” is the total number of seeds germinated in extract solutions, and “c” is the total number of seeds. Seeds were considered germinated when the radicle had protruded outside of the seed coat.

## 3. Results

### 3.1. The Dead Pericarps of Dry Fruits Accumulate Active Hydrolytic Enzymes

**Figure 1:**
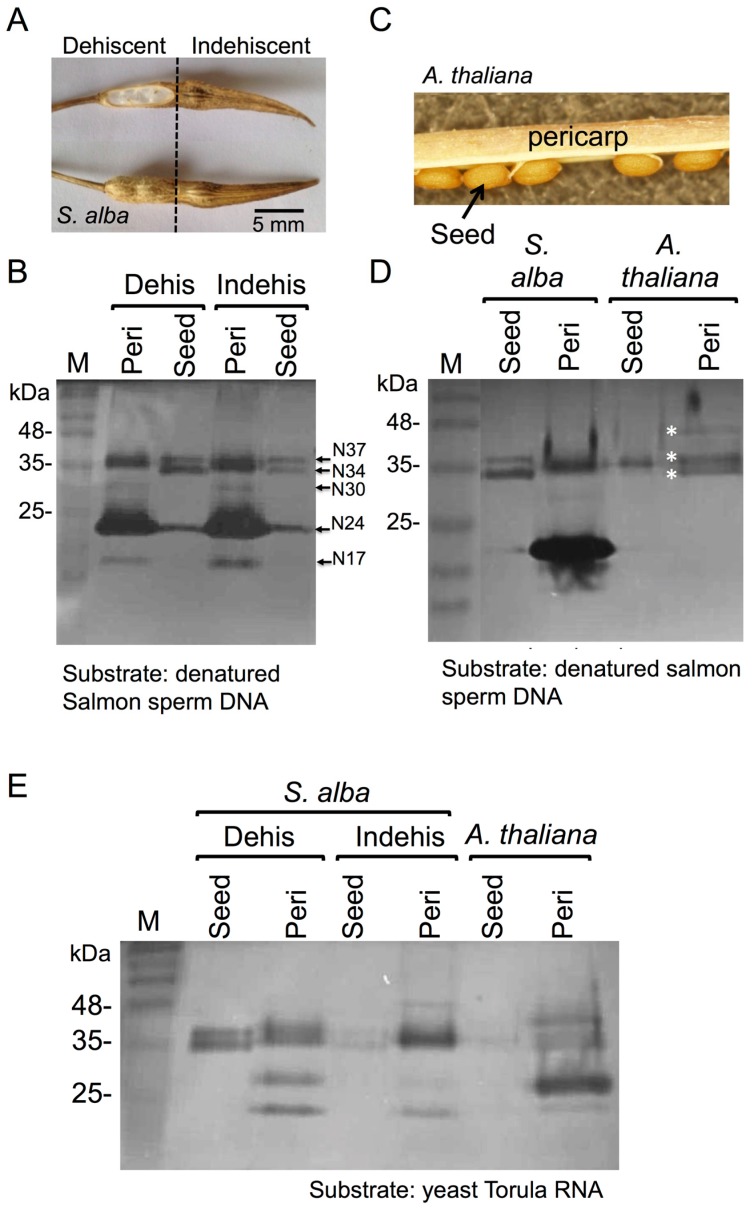
The dead pericarps of *S. alba* and *A. thaliana* releases active nucleases and RNAses. Ground pericarps derived from *S. alba* and *A. thaliana* were extracted with PBS buffer at 4 °C for 12 h and substances released were examined for nuclease activity. (**A**) Fruit of the *S. alba* dehiscent and indehiscent parts. (**B**) In gel nuclease assay demonstrating nuclease activity in dead dehiscent and indehiscent pericarps of *S. alba*. (**C**) Dehiscent fruit of *Arabidopsis thaliana*. Seeds and pericarp are indicated. (**D**) In gel nuclease assay demonstrating nuclease activity in pericarps of *A. thaliana*. Active nucleases are marked by white asterisks. (**E**) In gel RNase assay of proteins released from pericarps of *S. alba* and *A. thaliana*. M, protein molecular weight markers (in kDa).

In light of recent findings demonstrating that dead seed coat and floral bracts in grasses (e.g., glumes) function as storage for hydrolytic enzymes [17,18], we investigated the capacity of dead pericarps of dry fruits for storing and releasing hydrolytic enzymes upon hydration. To this end, *S. alba* fruit was selected for further study because it is composed of two distinguishable parts, dehiscent and indehiscent (Figure 1A). We examined *S. alba* dehiscent and indehiscent pericarps for nuclease activities by using in gel nuclease assay in the presence of Ca^2+^ and Mg^2+^ as cofactors in comparison with nucleases released from their corresponding seeds [18]. Results showed (Figure 1B) that both dehiscent and indehiscent pericarps store and release, following hydration, several nucleases at positions corresponding to 17 (N17), 24 (N24), 30 (N30), 34 (N34), and 37 (N37) kDa. Most notable activities were N24, N34, and N37 that occur also in substances released from *S. alba* seeds; N17 and N30 are evident in pericarp extract only, displaying very low activity. Similarly to *S. alba* pericarp, we could extract from the dead pericarps of *Arabidopsis thaliana* (Figure 1C) nucleases migrating to position of about 34, 37, and 40 kDa (asterisks Figure 1D). The analysis of RNases activities using in gel nuclease assay with yeast Torula RNA as substrate showed that dead pericarps of *S. alba* and *A. thaliana* possess multiple RNases ranging from 23 to 48 kDa (Figure 1E); the activities released from pericarps were higher than those released from seeds.

The dynamic of release of nucleases from pericarps was analyzed by repeated extraction with PBS. Ground pericarps were incubated in PBS buffer for 1 h at 4 °C, the supernatant was collected and analyzed by in gel nuclease assay. The results showed (Supplemental Figure S1) that in all pericarps examined including *S. alba* dehiscent and indehiscent pericarps (Figure S1A) as well as pericarps of *Coriandrum sativum* L. (Figure S1B, left panel) and *Hymenocarpus circinatus* (L.) Savi (Figure S1B, right panel) most nucleases were released in the first extraction round. Further re-extraction of pericarps showed the release of very low or no nuclease activity.

**Figure 2:**
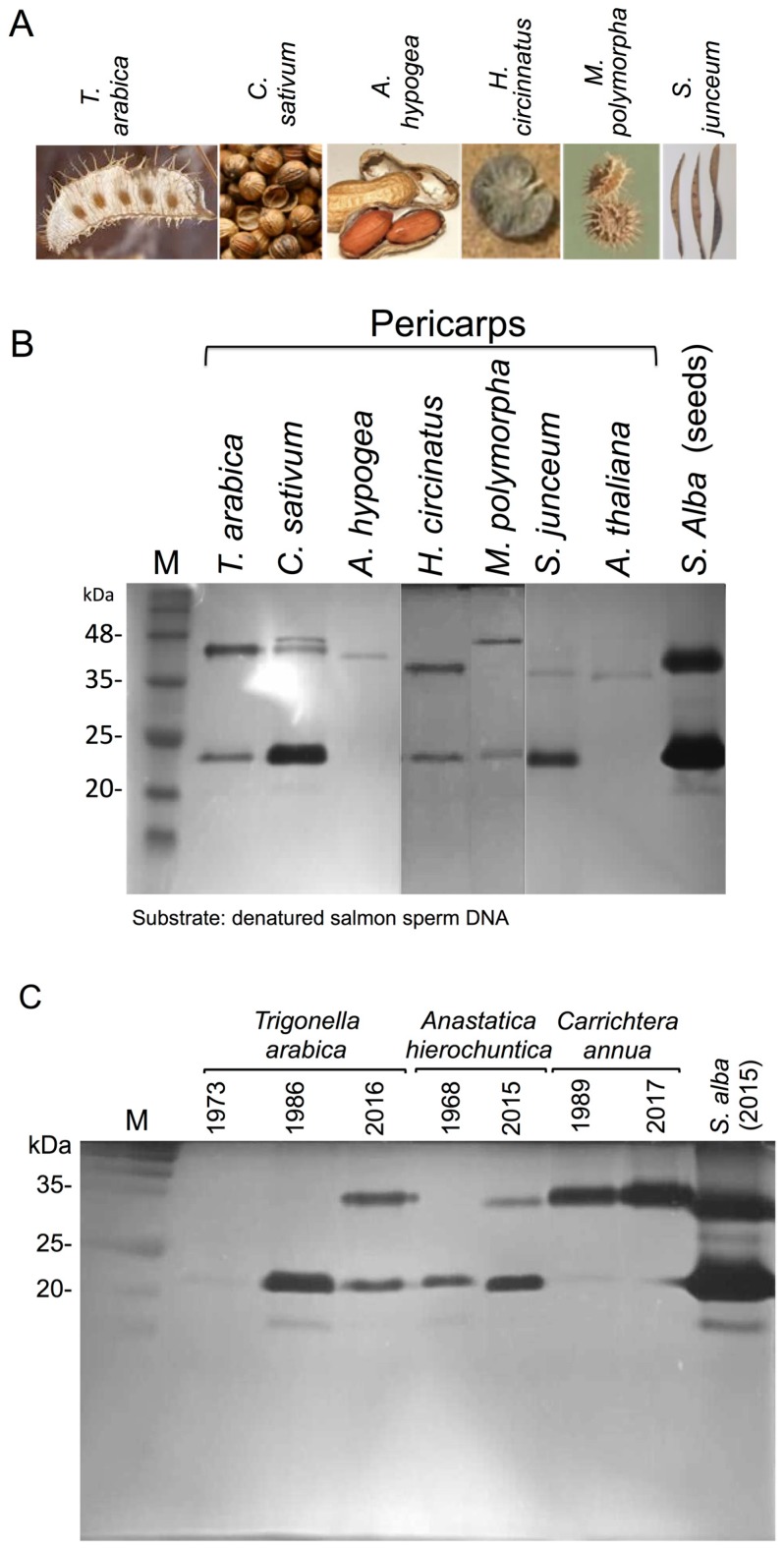
The dead pericarps of various plant species store and release upon hydration active nucleases. (**A**) Dry fruits of *Trigonella arabica*, *Coriandrum sativum*, *Arachis hypogea*, *Medicago polymorpha*, *Hymenocarpus circinnatus and Spartium junceum. (***B**) In-gel nuclease assay. Nuclease activity in pericarps of the above indicated plant species. *S. alba* seed secretion was used as a control with strong nuclease activity toward denatured salmon sperm DNA. M, protein molecular weight markers. (**C**) Long term persistence of active nucleases within dead pericarps—In-gel nuclease assay. Pericarps from indehiscent fruits of *T. arabica*, *Anastatica hierochuntica* and *Carrichtera annua* collected at the indicated year and stored at room temperature were analyzed for nuclease activity. *S. alba* pericarps collected from 2015 was used as a control with strong nuclease activity toward denatured salmon sperm DNA. M is the protein molecular weight markers.

To demonstrate the generality of protein storage in dead pericarps, we analyzed pericarps of dehiscent and indehiscent fruits of various plant species (Figure 2A) for nuclease activity including *Trigonella arabica* Delile (Fabaceae), *C. sativum* L. (Apiaceae), *Arachis hypogaea* L. (Fabaceae), *H. circinatus* (L.) Savi (Fabaceae), *Medicago polymorpha* L. (Fabaceae) and *Spartium junceum* L. (Fabaceae). Results showed (Figure 2B) that all examined dead pericarps possessed active nucleases ranging from 24 to 48 kDa.

We analyzed the persistence of nucleases within dead pericarps by analyzing various indehiscent dry fruits derived from *Trigonella arabica* (collected at 1973, 1986 and 2016), *Anastatica hierochuntica* (collected at 1968 and 2015) and *Callichtera annua* (collected at 1989 and 2017). All plant materials were stored at room temperature under the same conditions. Pericarps were isolated from the dry fruits, ground, extracted with PBS and released proteins were subjected to in gel nuclease assay. Results showed (Figure 2C) that except for *T. arabica* collected at 1973 all other pericarps (old and new collections) released highly active nucleases, demonstrating that nucleases can maintain their activity for decades during storage within the dead pericarps.

**Figure 3:**
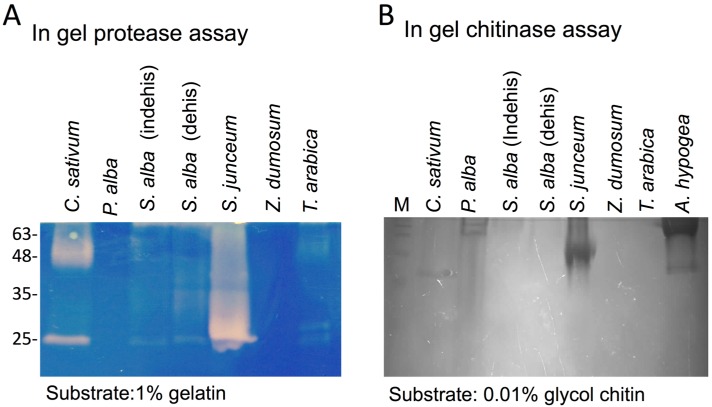
Dead pericarps of various plant species store and release proteases and chitinases. (**A**) In gel protease assay. Pericarps derived from *C. sativum, P. alba*, *S. alba*, *S. junceum*, *Z. dumosum* and *T. arabica,* were extracted (10 mg) with 100 μL of PBS buffer at 4 °C for 12 h and substances released were examined for protease activity toward 1% gelatin. (**B**) Detection of active chitinases by in gel chitinase assay. Pericarps of *C. sativum*, *P. alba*, *S. alba*, *S. junceum*, *Z. dumosum*, *T. arabica* and *A. hypogea* were extracted (10 mg except that 1 mg was used for *S. junceum*) with 100 μL of 0.1 M NaHPO_4_ (pH = 6) at 4 °C for 8 h and substances released were examined for chitinase activity towards 0.01% glycol chitin. M, molecular weight protein markers (in kDa).

Pericarps derived from various species also displayed protease (Figure 3A) and chitinase (Figure 3B) activities as demonstrated by in gel assays. Strong protease and chitinase activities were evident in *S. junceum* pericarps; strong protease activity was also apparent in *C. sativum* pericarps.

### 3.2. Proteome Analysis of S. alba Indehiscent Pericarps

**Figure 4:**
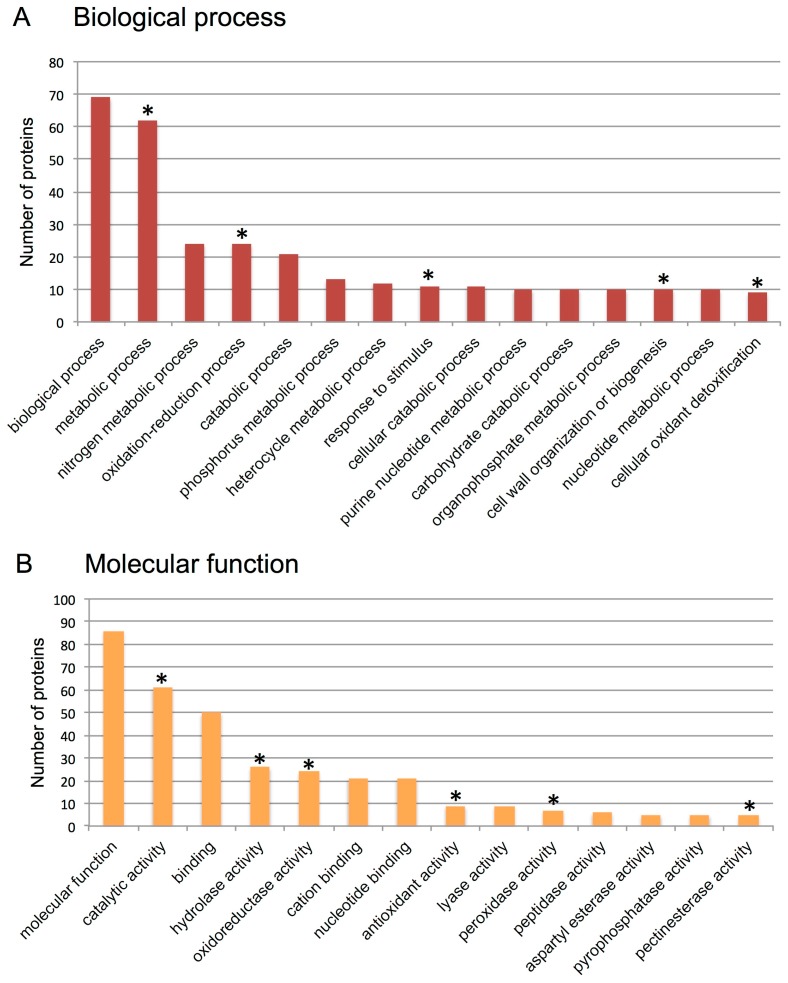
Analysis of proteins released from pericarps of *S. alba* (proteome analysis). Gene Ontology (GO) categorization for biological process (**A**) and molecular function (**B**). Asterisks indicate overrepresented protein groups in the pericarp samples. Note, the first column in A and B indicates the number of proteins identified for the examined categorization. Also, other columns do not add up to the first one because a given protein can fit multiple sub-categorization.

Next we sought to identify proteins accumulated in indehiscent dry fruit of *S. alba* (i.e., the beak) using proteome analysis. Applying the cutoffs (10% coverage) described in Materials and Methods, 101 proteins that are considered present were identified. Functional categorization showed that among the 69 proteins recognized in biological process category, 62, 24 and 11 proteins are involved in metabolic, oxidation-reduction and response to stimuli, respectively (Figure 4A). In addition we found that among the 86 proteins recognized in molecular function, category 61 have catalytic activity, while 24 and 26 proteins have oxireductase and hydrolase activities, respectively. Of particular interest was the finding of overrepresentation of proteins involved in cell wall modification and biogenesis such as pectinesterases and polygalacturonases (Supplementary Table S2) as well as in reactive oxygen species (ROS) detoxification (antioxidant activity) including superoxide dismutases and peroxidases (Supplementary Table S3).

### 3.3. The Dead Pericarps of Arabidopsis and Sinapis Accumulate Nutrients

**Figure 5:**
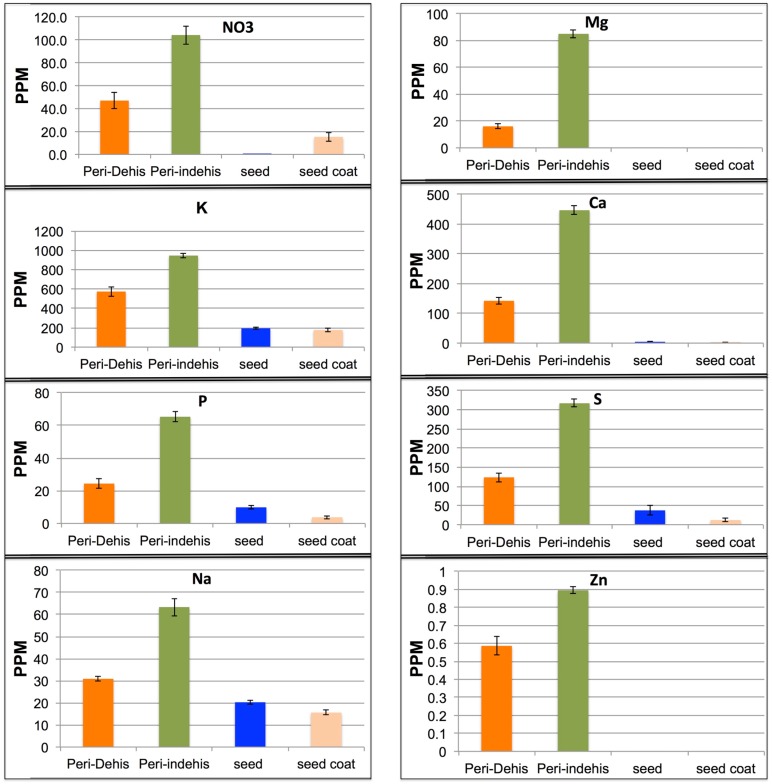
High levels of nutrients are stored and released from dead pericarps of *S. alba* upon hydration. Extracts derived from intact seeds, seed coats, dehiscent pericarps (Peri-Dehis) and indehiscent pericarps (Peri-indehis) were subjected to nutrient detection by ICP-OES. The concentration (ppm) of each element examined is shown. Bars represent the standard error.

We used the Inductively Coupled Plasma (ICP) for element analysis in extracts of pericarps derived from *S. alba* dehiscent and indehiscent fruit parts and *Arabidopsis* (Ler ecotype) dead pericarps. We compared elemental analysis of the pericarps with the elements released from intact seeds or from seed coat only (in the case of *S. alba*). Results showed that the pericarps of *S. alba* (Figure 5) and of *Arabidopsis* (Supplementary Figure S2) release large amounts of nutrients compared to seeds and seed coats. In *S. alba* pericarps, some elements were accumulated at high levels, particularly in the indehiscent pericarp including K (945 ppm), Ca (446 ppm), S (316 ppm) Mg (85 ppm), and NO${}_{3}^{-}$ (nitrate, 104 ppm) (Figure 5). Similarly, we detected a large amount of nutrients in extracts of *Arabidopsis* pericarp including K (1532 ppm), Ca (840 ppm), S (115 ppm), and Mg (139 ppm) and NO${}_{3}^{-}$ (nitrate, 615 ppm) (Supplementary Figure S2). Thus our results highlighted dead pericarps as a rich source for nutrients that can potentially used for seedling nourishment.

### 3.4. Effects of Pericarps on S. alba Seed Germination

Although the effect of dehiscent and indehiscent pericarps on *S. alba* seed germination in petri dishes was reported previously [25], we wanted to examine germination under field conditions. The germination results from field experiments confirmed Sroelov [25] experiments in petri dishes showing that seeds enclosed within indehiscent fruit (i.e., beak) showed no germination compared to naked indehiscent seeds (50% germination, Supplementary Figure S3A). Moreover, dehiscent seeds were almost fully germinated (94%) but showed reduction in germination (61%) in the presence of dehiscent pericarps (Supplementary Figure S3A), suggesting that pericarps contain germination inhibitory substances that can act under field growth conditions. Germination experiments conducted in petri dishes showed similar results as field experiments with no germination from the indehiscent beak, compared to almost full germination from naked seeds (Supplementary Figure S3B).

**Figure 6:**
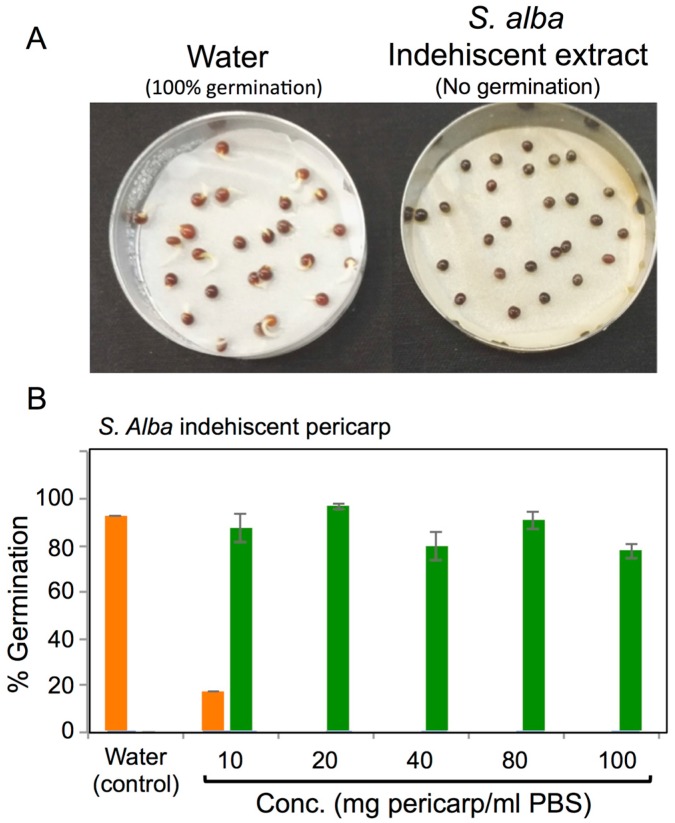

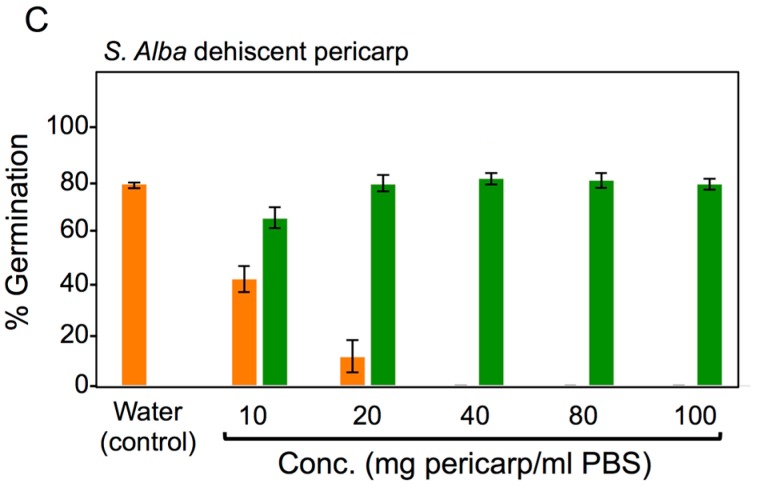
Dehiscent and indehiscent pericarps of *S. alba* contain germination inhibitory substances-Lab experiments. (**A**) Germination test of *S. alba* seeds incubated in distilled water or in *S. alba* indehiscent pericarp extract. Note that there is complete inhibition in the presence of indehiscent pericarp extract. Seeds of *S. alba* were incubated in distilled water and different concentrations (10, 20, 40, 80, 100 mg pericarp/mL of distilled water) of indehiscent (**B**) and dehiscent (**C**) *S. alba* pericarp extracts. After 56 h, the seeds treated with pericarp extracts were washed thoroughly with water and seeds were incubated in water for assessment of germination recovery. Note, seed germination was reduced significantly in the presence of pericarp extracts (orange column) but fully recovered after removal of extracts and incubation in water (green column). Data are the mean of three replicates (±SE).

Next, we examined the effect of extracts derived from dehiscent and indehiscent pericarps on *S. alba* seed germination in petri dishes and the potential recovery of germination. Incubation of *S. alba* seeds in extracts derived from indehiscent pericarps completely inhibited germination compared to full germination obtained under water treatment (Figure 6A). Detailed analysis of dehiscent and indehiscent pericarp extracts on germination revealed that both have a strong inhibitory effect on germination, which is dependent on the extract concentration (Figure 6B,C, orange column). The indehiscent pericarp extract had a stronger effect inasmuch as it completely inhibited germination at a relatively low concentration of 20 mg/mL compared to dehiscent pericarp that showed complete inhibition only in 40 mg/mL. The recovery of germination was recorded for each treatment after non-germinated seeds were rinsed three times with 10 mL of distilled water and then placed in new petri dishes with the filter paper moistened with distilled water and incubated for three more days. The results (Figure 6B,C green columns) revealed the *S. alba* seeds could completely recovered from all treatments including the higher extract concentration (100 mg/mL) of dehiscent and indehiscent pericarps. Thus, our results demonstrated that substances released from the *S. alba* pericarps contain strong germination inhibitors but once they are washed away, the seeds germinate normally.

To examine the specificity of the germination inhibitors stored and released from *S. alba* pericarps, we evaluated the germination of *B. rapa* seeds (close relative of *S. alba*) in the presence of *S. alba* pericarp extracts. We found that *B. rapa* seeds were inhibited under high concentrations of extracts derived from indehiscent pericarps (Supplemental Figure S4A). Accordingly, indehiscent pericarps showed no inhibition at concentrations up to 40 mg/mL. However at higher concentrations (80 and 100 mg/mL) a notable inhibition of about 50% is evident. At high concentrations, dehiscent pericarps showed only slight reduction of *B. rapa* seed germination (Supplemental Figure S4B). Thus the effect of germination inhibitory factors derived from *S. alba* pericarps on germination of *B. rapa* seeds is essentially similar to its effect on germination of *S. alba* seeds, except that higher concentrations are required for execution of inhibition.

### 3.5. S. alba Pericarp Extracts Promote Bacterial Growth

**Figure 7:**
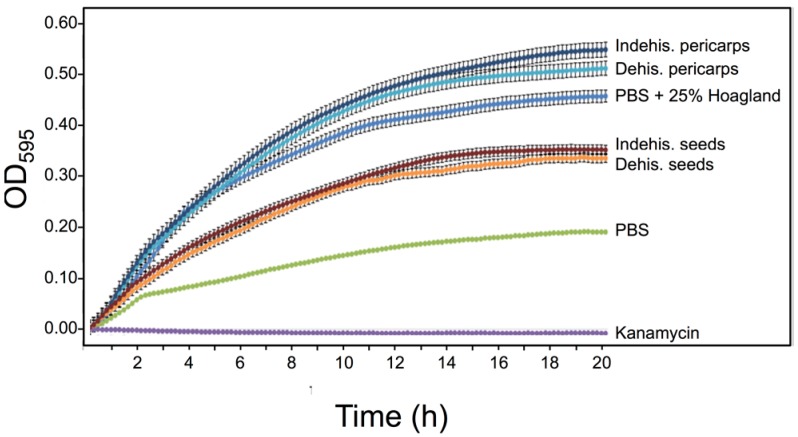
Pericarps of *S. alba* dehiscent and indehiscent fruit parts release substances that promote bacterial growth. *Escherichia coli* was grown in a flat-bottom 96-well microtiter plate in the presence of PBS, PBS + 25% Hoagland solution, kanamycin (50 μg/mL), or in the presence of substances released from dehiscent and indehiscent seeds or pericarps. Bacterial growth was monitored by measuring the OD_595_ of the culture at 30 min. intervals in the course of 20 h. Each treatment was performed in triplicates and error bars represent the standard deviation.

Microorganisms have a range of inhibitory and stimulatory effects on early seed germination and seedling growth [26]. We wanted to examine for the presence of microbial growth controlling activity in substances released from dehiscent and indehiscent pericarps of *S. alba*. We used the gram-negative strain *E. coli* for our experiment. Bacteria were grown in a flat-bottom 96-well microtiter plate in LB medium supplemented with PBS, kanamycin, Hoagland solution (25%), pericarp extracts or seed secretions derived from *S. alba*. Plates were incubated in the dark using a Synergy 4 spectrophotometer (Biotek, USA) and reads (OD_595_) were taken at 30 min intervals in a course of 20 h. Growth of *E. coli* was strongly enhanced when incubated in extracts derived from dehiscent and indehiscent pericarp (Figure 7) compared to PBS or to *S. alba* seed secretions. The promoting effect may be related, at least partly, to the nutrient content inasmuch as the bacterial growth was enhanced compared to PBS only in the presence of nutrients (PBS + 25% Hoagland).

## 4. Discussion

The pericarp (indehiscent dry fruits) is commonly considered to be a passive barrier functioning in embryo protection and seed germination, as well as providing the means for seed dispersal. Here we uncovered a previously unrecognized function of dead pericarps of both dehiscent and indehiscent fruits in storing active hydrolytic enzymes and other substances that are released upon hydration and might play important roles in regulating seed longevity, germination, and seedling establishment. The present data together with previous reports [17,18] suggest that storage of proteins and other substances within dead organs enclosing embryos is a general theme in plant reproductive biology that have been evolved to increase the success of germination and seedling establishment.

Here we showed that dehiscent and indehiscent pericarps possess highly active DNases acting toward single stranded DNA (e.g., endonucleases) and RNases. Notably, multiple nucleases involved in seedling development were characterized in cauliflower whose expression was induced by drought stress and hydrogen peroxide [27]. Presently, the role played by nucleases in seed germination and seedling establishment is unknown. Generally, endonucleases appear to be involved in multiple cellular processes including DNA synthesis and repair [28], as well as in fragmentation of genomic DNA during cell death [29,30,31]. The findings that endonucleases are capable of introducing nicks and double strand DNA breaks into superhelical DNA [32,33] suggest a possible role defense against plasmid-bearing soil pathogens such as the *Clavibacter michiganensis* subsp. *michiganensis* [34]. Also, multiple active RNases identified in extracts of *Arabidopsis* and *S. alba* pericarps may act as pathogen inhibitors [35,36,37]. In-gel assays also revealed chitinases, enzymes that degrade chitin, are often over-expressed in plants to confer resistance against fungal pathogens [38,39,40]. Proteases are believed to function in degrading proteins to provide amino acids for protein synthesis within the embryo [41]. Proteases, however, play an important regulatory role in diverse biological processes including cell cycle progression, embryogenesis, cell fate determination, defense response, and seed coat formation [42]. Proteolytic enzymatic activity can target and degrade essential proteins that could lead to death of cells, tissues, organs and even the whole organism. Thus, proteases could provide an important protective layer for defending germinating seeds from soil pathogen.

Besides the potential of combating soil pathogens by hydrolytic enzymes, *S. alba* pericarps, similarly to *S. alba* seed coats [18], release substances that can promote microbial growth. Notably, growth promotion of beneficial soil-borne microbes by substances released from the pericarp might contribute to plant growth and development. Plant growth-promoting bacteria can influence root development, enhance plant growth by various means including increasing nutrient availability [e.g., nitrogen (N), iron (Fe), and phosphorus (P)] and production of indolic compounds [e.g., Indole-3-acetic acid (IAA)] as well as by protecting plants from diseases, at least partly by suppressing deleterious soil-borne pathogens [43,44,45]. Thus, accumulation of microbial growth-controlling substances in maternally derived dead tissues of pericarps and seed coats might act directly or indirectly to control soil-borne microbes. We hypothesize that the composition of microbe growth controlling substances could be affected by the conditions to which mother plants are exposed to during flowering and seed development, a topic currently studied in the lab.

The proteome data revealed 101 proteins released from the indehiscent pericarps of *S. alba* including proteases and chitinases. Of particular interest were two groups of proteins that might have crucial rule in germination including ROS detoxifying enzymes and cell wall modification enzymes. 

ROS play an important role in various aspects of seed biology [46,47]. They are produced during various stages of seed development, including seed maturation and desiccation, storage (aging) and seed germination. ROS production during seed development could culminate in oxidative stress-damaging macromolecules such as proteins and DNA, which might lead to seed deterioration. This highlighted the importance of ROS “detoxifying” enzymes such as superoxide dismutases (SODs), catalases, and peroxidases, as well as other antioxidants in keeping the appropriate balance of ROS in the cell, and consequently seed viability. While ROS has long being considered as toxic molecules there is a growing body of evidence implicating ROS also as signaling molecules playing important roles in releasing seed dormancy and seed germination as well as providing a defense against soil pathogens [46,47,48,49].

Thus, it is possible that these enzymes are released upon hydration to the immediate surrounding of the germinating seed to fulfill multiple functions. One is to ensure that seed germination microenvironment is free of hazardous radicals that could harm the germinating seed (protruding radicles), or to allow for the generation of specific radicals, which are important for seed germination on the one hand, and on the other hand are necessary to combat potential pathogens.

The overrepresentation of pectinesterases (PEs) and polygalacturonases (PGs) in indehiscent pericarps suggest a role in modifying and softening cell walls to allow for the radicle to protrude outside the pericarp. Both PEs and PGs are pectinases that break down pectin—the major constituent of plant cell walls. They affect various aspects of plant growth and development through their effect on the integrity of cell walls. PEs and PGs were reported to affect the mechanical stability of cell walls during fruit ripening, involved in cell wall loosening of the endosperm necessary for radicle protrusion, cell wall extension during pollen germination and pollen tube growth, abscission as well as stem elongation [50,51].

Substances released from pericarps of *S. alba* have the potential to inhibit seed germination, a phenomenon described previously for *S. alba* pericarps in vitro [25]. Here, we further showed that pericarps exert similar effects under field growth conditions. Pericarps of various plant species were implicated in the inhibition of seed germination by various means, including reducing water uptake by seeds, releasing germination inhibitors or mechanically by restricting embryo growth and preventing radicle protrusion [10,11,12,13]. In her report, Sroelov [25] showed that the beak, valves, and pedicel of *S. alba* contain germination inhibitors, which are thermostable and water soluble. We also showed that inhibition of seed germination was relieved as soon as the pericarp extract was removed and seeds washed and re-incubated in water. Thus, *S. alba* dehiscent and indehiscent pericarps release water soluble germination inhibitors whose chemical structure are presently unknown. Germination inhibitors were identified in glumes and hull of *Aegilops kotschyi* to act via a gibberellin-inhibiting mechanisms and have coumarin- or abscisin-like activity [52].

Of particular interest was the finding of accumulation of high levels of nutritional elements within the dead pericarps of *Arabidopsis* and *Sinapis* compared to the seed or the seed coat, including nitrate, potassium, phosphorus, and sulfur. Several studies have shown that nutrients such as potassium, calcium and other cations are stored in the bracts of wild wheat and *Eurotia*, and can move into the seed during imbibition [17,53]. Thus, the dead organs enclosing seed/caryopsis might serve as an immediate nutritional supply for germinating seeds. Germination assays performed to assess the importance of the dead floral bracts enclosing the caryopsis in wild emmer wheat revealed an apparent advantage of germination from the intact dispersal unit (DU) compared to naked caryopsis. Particularly, DU-seedlings displayed higher number and length of lateral roots compared to naked seed seedlings, which could be accounted for the presence of high levels of nutritional elements (NO_3_, K, P, and S) released from dead floral bracts upon hydration [17]. Indeed, in barley, exogenous supply of nitrate or phosphate led to increase in lateral root growth [54]. Multiple reports highlighted potassium, nitrate, and potassium nitrate (KNO_3_) as important factors contributing to seed germination, as well as to rapid root growth and seedling establishment [55,56,57,58,59,60]. Potassium is a major essential nutrient for plant growth and development, which is commonly accumulated to high levels often constitutes between 2–10% of plant dry weight [61,62]. Importantly, potassium plays many important regulatory roles in plant development where it is required for multiple plant growth processes, including enzyme activation, photosynthesis, and protein synthesis. Also, potassium plays an important role in defending plants against biotic and abiotic stresses, including diseases, pests, drought, salinity, cold, frost, and waterlogging [63]. Thus, the nutritional supply embedded within the dry, dead pericarps provide another versatile layer for nourishing the growing embryos and assisting seedling establishment as well a defense layer against biotic and abiotic stresses.

## 5. Conclusions

Storage of proteins and other substances within dead organs enclosing embryos, including seed coats, pericarps and floral bracts in grasses, appears to be a general theme in plant reproductive biology, providing embryos with multiple means (e.g., nutrition, growth factors and defense proteins) for increasing establishment success in the ecological niche.

A common practice in agriculture for enhancing seed performance is through the addition of chemicals (coating) to protect the germinating seed from pathogens, as well as adding other substances for increasing germination success. Our data suggest that pericarps of dry fruits should be viewed as “natural coatings” capable of “engineering the microenvironment” for the benefit of the embryo. This should trigger discussion regarding (i) the way we treat plant dead organs in various agricultural practices, (ii) the way we store plant genetic resources (ex situ conservation) in seed banks for future usage, (iii) changing agricultural practices for better and healthier environments by replacing hazardous chemical coating of seeds with “green”, and non-hazardous coatings derived from dead organs enclosing embryos.

## Supplementary Materials

The following are available online at www.mdpi.com/2223-7747/6/4/64/s1, Supplemental Data 1: Figure S1. Dynamics of nuclease release from the dead pericarps. Figure S2. Nutrient analysis of A. thaliana pericarps. Figure S3. Field germination experiments. Figure S4. Effect of *S. alba* pericarp extract on *B. rapa* seed germination. Table S1. List of plant species used in this study. Table S2. Pectinesterases and polygalacturonases released from *S. Alba* indehiscent pericarps (proteome analysis). Table S3. ROS detoxifying enzymes released from *S. Alba* indehiscent pericarps upon hydration (proteome analysis). Supplemental data 2. Proteome raw data of proteins released from *S. alba* indehiscent pericarps.
