# Supplementary material for: Dead Pericarps of Dry Fruits Function as Long-Term Storage for Active Hydrolytic Enzymes and Other Substances That Affect Germination and Microbial Growth
Source: Plants (Basel). 2017 Dec 19;6(4):64. doi: 10.3390/plants6040064 (PMC5750640; doi:10.3390/plants6040064)
Supplement: Supplementary file 1 [file plants-06-00064-s001.zip › Supplemental data 1.pdf]

Supplementary Materials

Dead Pericarps of Dry Fruits Function as Long-Term Storage for Active Hydrolytic Enzymes and Other Substances That Affect Germination and Microbial Growth

Godwin James, Buzi Raviv and Gideon Grafi *

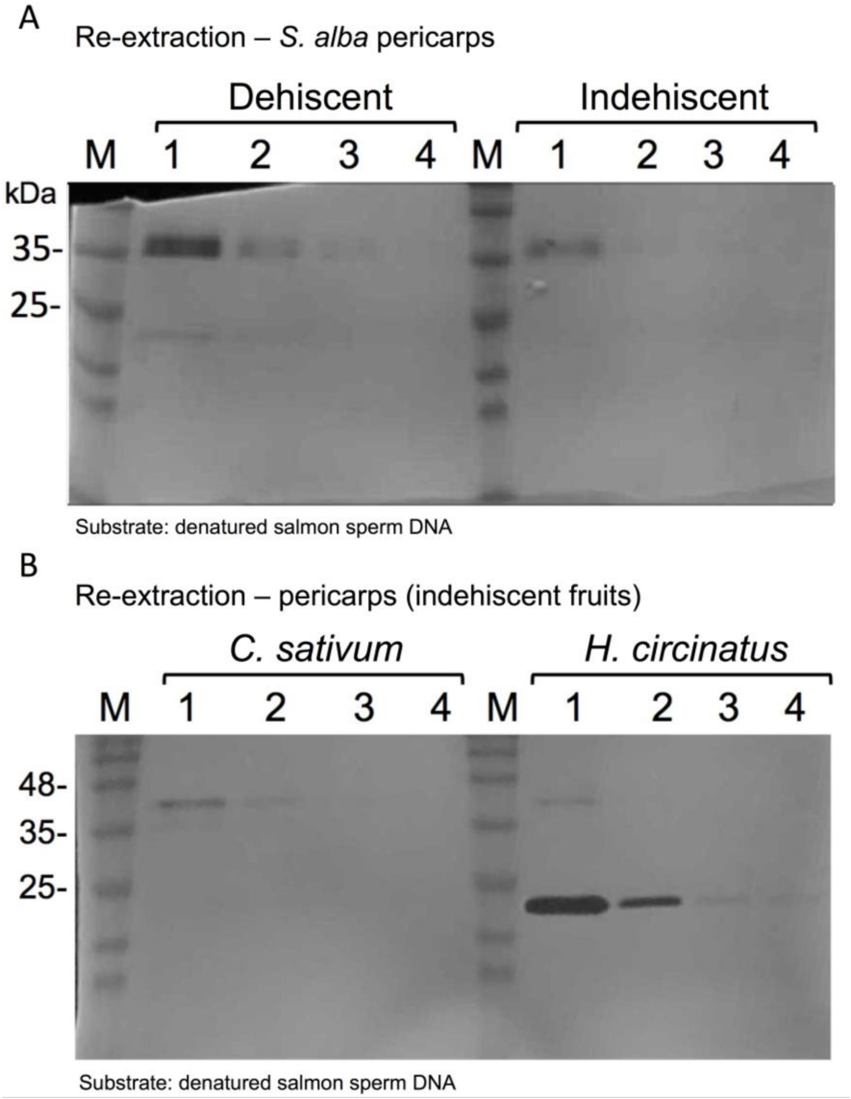

Figure S1. Dynamics of nuclease release from the dead pericarps. Pericarps were subjected to four repeated extraction with PBS (1-4), each extraction was for 1 h at 4 ℃ after which the supernatant was collected and analyzed for nuclease activity using in gel assay with denatured salmon sperm DNA. (A) Repeated extraction of *S. alba* dehiscent and indehiscent pericarps. (B) Repeated extraction of nucleases from indehiscent pericarps of *C. sativum* and *H. circinatus.* M, molecular weight protein markers.

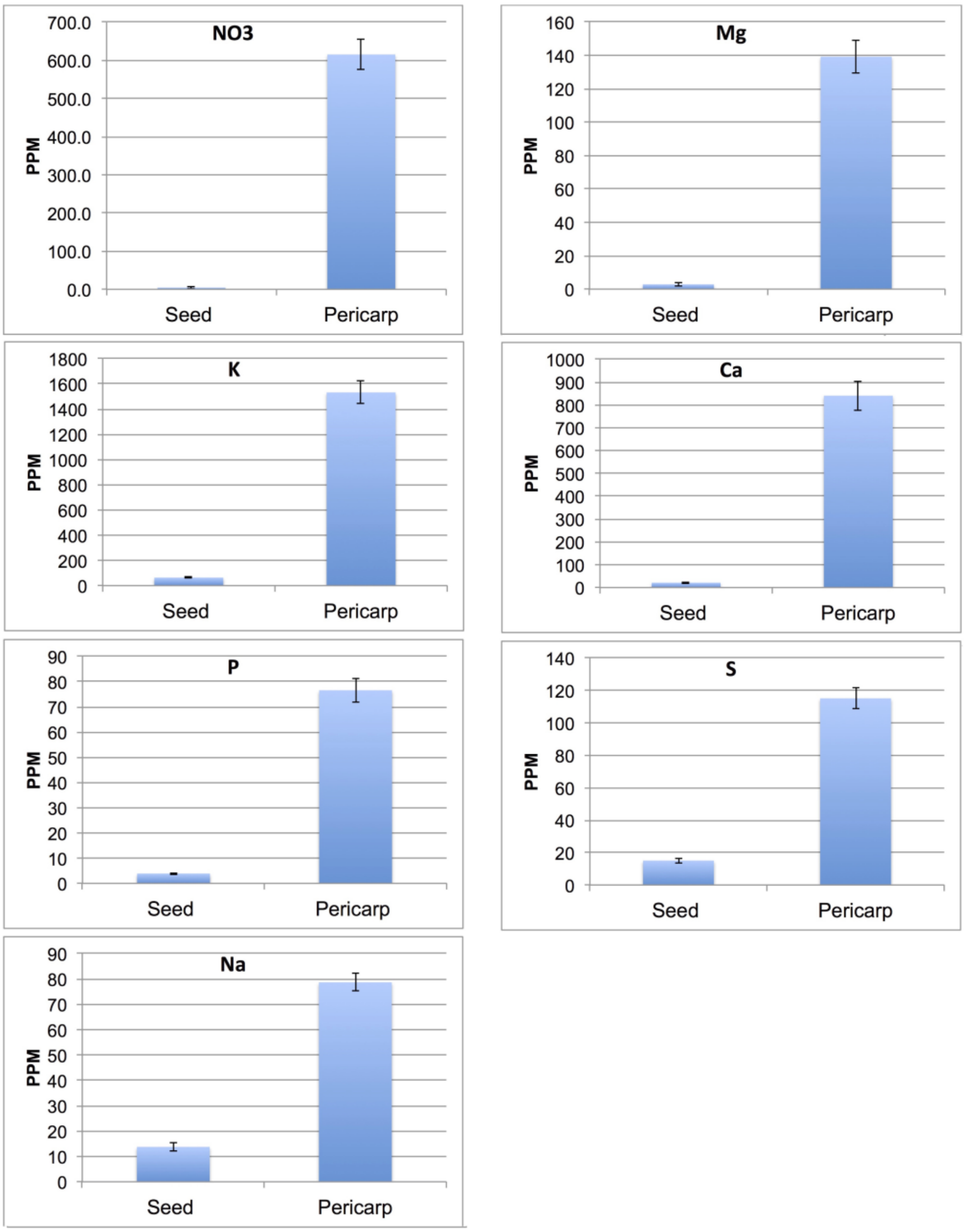

Figure S2. High levels of nutrients are stored and released from dead pericarps of *A. thaliana* upon hydration. Intact seeds and dehiscent pericarps were incubated in 0.8 ml PBS at 4°C for 12 h, the sup was collected and subjected to nutrient detection by ICP-OES. The concentrations (ppm) of each element detected are shown. Bars represent the standard error calculated from three measurements.

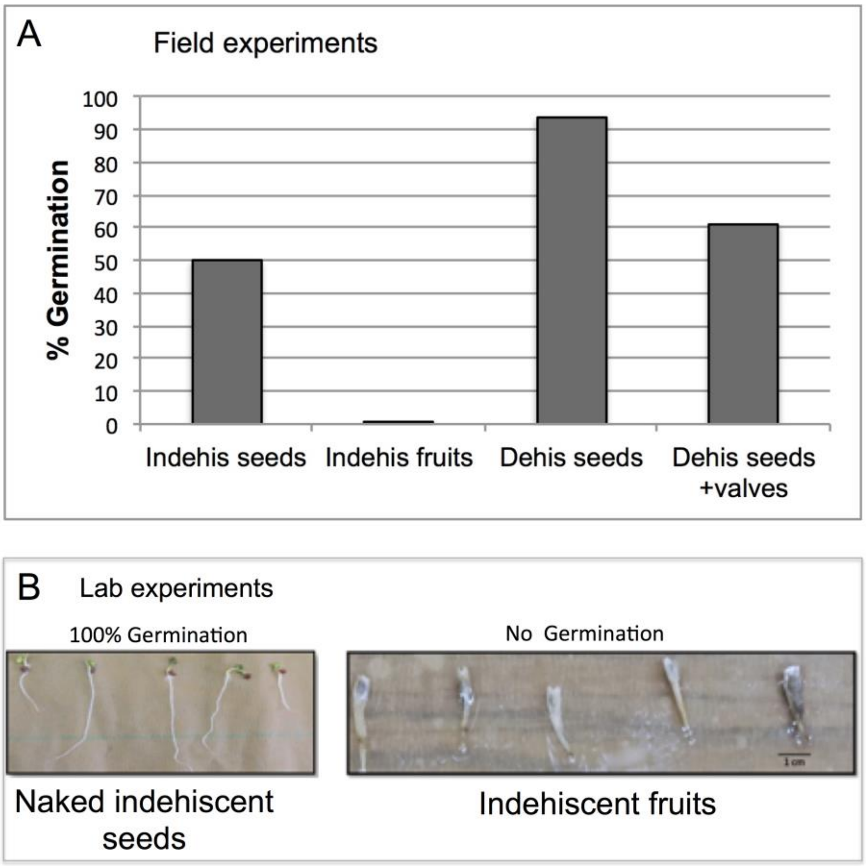

Figure S3. Dehiscent and indehiscent pericarps of *S. alba* contain germination inhibitory substances – Field experiments. (**A**) Field experiments were performed on loessial soil in the northern Negev (31°26’57” N / 34°39’31” E, elevation 176 m) using a 36 square grid (4x9), each square is 3x3 cm. 18 seeds or seeds with pericarps or 18 beaks containing seeds of *S. alba* were placed in a 36 square grid on loessial soil in northern Negev and germination was scored after 3 months. Note no germination observed for indehiscent fruit part of *S. alba.* (**B**) In vitro germination of naked indehiscent seeds versus indehiscent fruits.

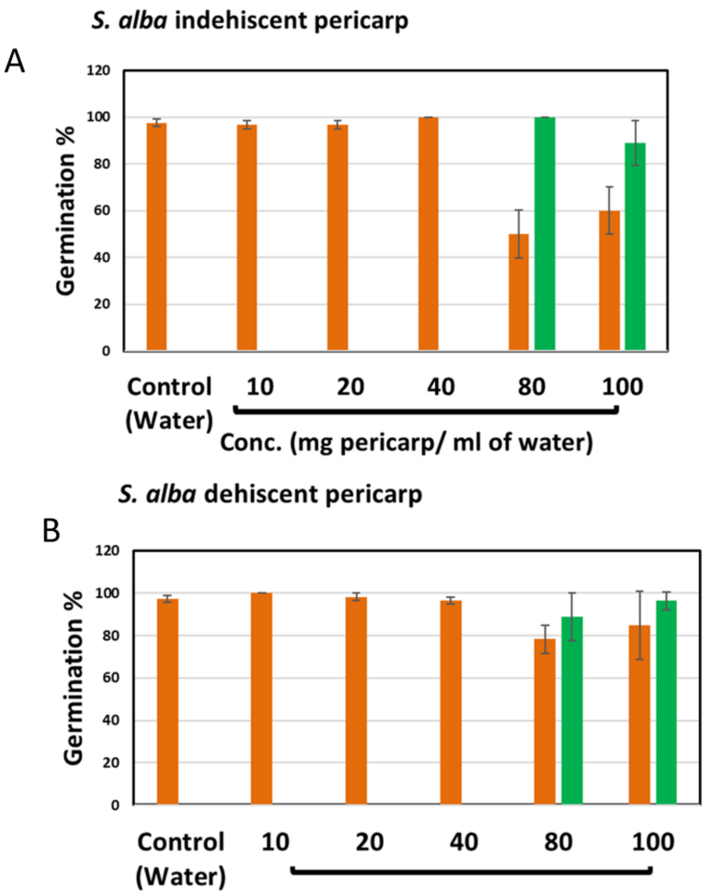

Figure S4. Extracts derived from indehiscent pericarp of S. *alba* inhibit germination of *Brassica rapa* seeds. (**A**) Effects of indehiscent pericarp extracts on *Brassica rapa* germination. (**B**) Effects of dehiscent pericarp extracts on *Brassica rapa* germination. Seeds of *B. rapa* were incubated in distilled water and different concentrations (10, 20, 40, 80, 100 mg pericarp/ ml of distilled water) of *S. alba* pericarp extracts. After 24 hours, the seeds treated with pericarp extracts were washed thoroughly with water and seeds were incubated for recovery germination in water. Note, seed germination was not inhibited in the presence of pericarp extracts (orange column) but fully recovered after removal of extracts (concentration 80 and 100 mg/ml) and incubation in water (green column). Data are the mean of three replicates (±SE).

Table 1. A list of plant species present in this study.

Species name	Family	Collection year	Plant type	Life form	Place of collection
Arabidopsis thaliana (L.) Col-0	Brassicaceae	2010	Common lab line	Annual	Lab line
Sinapis alba (L.) Beassicaceae	Brassicaceae	2014	Wild plant	Annual	Northern Negev/ side roads
Hymenocarpus circinatus (L.) Savi	Fabaceae	2016	Wild plant	Annual	Northern Negev/ side roads
Spartium junceum (L.)	Fabaceae	2017	Ornamental	phanerophyte shrub	Northern Negev / local garden
Trigonella arabica Delile	Fabaceae	1973, 1986, 2016	Wild plant	Annual	Negev/Midreshet Ben Gurion
Coriandrum sativum L.	Apiaceae	2016	Cultivated	Annual	Local market
Arachis hypogaea L.	Fabaceae	2016	Cultivated	Annual	Local market
Medicago polymorpha L.	Fabaceae	2016	Wild plant	Annual	Negev/Midreshet Ben Gurion
Prosopis alba Grisebach	Fabaceae	2017	Ornamental	Perennial tree	Negev/Midreshet Ben Gurion
Anastatica hierochuntica	Brassicaceae	1968, 2017	Wild plant	Annual	Sinafir islands, Dead- Sea valley
Carrichtera annua	Brassicaceae	1989, 2017	Wild plant	Annual	Negev/Midreshet Ben Gurion

Table S2. A list of pectinesterases and polygalacturonases released from S. Alba indehiscent pericarps upon hydration (proteome analysis).

Accession	Description
XP_018472786.1	pectinesterase/pectinesterase inhibitor 17 [Raphanus sativus]
XP_018440051.1	pectinesterase/pectinesterase inhibitor 17 [Raphanus sativus]
XP_013675925.1	pectinesterase/pectinesterase inhibitor 3-like [Brassica napus]
XP_013675925.1	pectinesterase/pectinesterase inhibitor 3-like [Brassica napus]
XP_018440779.1	pectinesterase/pectinesterase inhibitor 3-like [Raphanus sativus]
XP_013644096.1	pectinesterase/pectinesterase inhibitor 3-like [Brassica napus]
XP_013622811.1	pectinesterase/pectinesterase inhibitor 3-like [Brassica oleracea var. oleracea]
XP_018467447.1	pectinesterase/pectinesterase inhibitor 18, partial [Raphanus sativus]
XP_013664232.1	probable pectinesterase/pectinesterase inhibitor 44 isoform X5 [Brassica napus]
XP_009116452.1	polygalacturonase ADPG1 isoform X2 [Brassica rapa]
XP_018444632.1	polygalacturonase ADPG1 [Raphanus sativus]
XP_018489829.1	polygalacturonase ADPG1 [Raphanus sativus]

Table S3. A list of ROS detoxifying enzymes released from *S. Alba* indehiscent pericarps upon hydration (proteome analysis).

Accession	Description
AAD05576.1	Cu/Zn superoxide dismutase [Raphanus sativus]
XP_018453774.1	superoxide dismutase [Fe] 1,[Raphanus sativus]
ACT35470.1	peroxidase 12, partial [Brassica rapa]
P00434.3	Peroxidase P7; [PERP7_BRARR]
XP_018483754.1	peroxidase 47 [Raphanus sativus]
XP_018460650.1	peroxidase 34 [Raphanus sativus]
XP_013606980.1	peroxidase 54 [Brassica oleracea var. oleracea]
XP_018463254.1	peroxidase 34-like [Raphanus sativus]
XP_013646868.1	peroxidase 34-like isoform X1 [Brassica napus]
